# Special Issue “Beneficial and Detrimental Microorganisms Occurring in Fermented Foods”: Editorial

**DOI:** 10.3390/microorganisms11030565

**Published:** 2023-02-24

**Authors:** Vincenzina Fusco, Hikmate Abriouel, Evandro Leite de Souza

**Affiliations:** 1 Institute of Sciences of Food Production of the National Research Council of Italy (CNR-ISPA), 70126 Bari, Italy; 2 Área de Microbiología, Departamento de Ciencias de la Salud, Facultad de Ciencias Experimentales, Universidad de Jaén, 23071 Jaén, Spain; 3 Department of Nutrition, Health Sciences Center, Federal University of Paraíba, João Pessoa 58051-900, Brazil

Numerous and heterogeneous populations of beneficial microorganisms originating from raw materials, equipment, and production and processing environments can affect the fermentation process by their metabolic activities, allowing for the enhancement of the nutritional value, sensory characteristics, overall quality, safety, and shelf-life of final food products [1,2,3]. In addition to the beneficial technological microorganisms, probiotic microorganisms or living microorganisms genetically similar to strains used as probiotics may occur in fermented foods, which may provide health benefits beyond those of the starting food materials [4,5,6].

On the other hand, multiple sources of the contamination of raw materials, equipment, and environments involved in the manufacturing of fermented foods may allow for the establishment and proliferation of spoilage and pathogenic microorganisms, which can cause alterations in the final products and threaten consumer health [2,7].

This Special Issue of *Microorganisms*, dedicated to “Beneficial and Detrimental Microorganisms Occurring in Fermented Foods”, aimed at collecting new studies concerning any aspect of pro-technological, probiotic, spoilage, and/or pathogenic microorganisms occurring in fermented foods, as well as on the characterization, evolution, and metabolism of microbiota that occurs during the production, storage, and distribution of these products.

In their study, Hussain et al. [8] examined the bacterial composition of fermented fruit products and evaluated their safety using high-throughput 16S-rRNA metagenetic analysis.

Jeong et al. [9] investigated the quality of traditionally made *donejang*, a fermented soybean product, using 29 samples originating from different regions of Korea. In particular, the biogenic amine content, bacterial composition, and metabolic functions were assessed.

Fagbemigun et al. [10] investigated by culture-dependent and -independent methods the microbial diversity of nono, a traditional fermented dairy food produced from cow’s milk in Nigeria, and selected a potential starter culture to be used to improve the microbial safety and quality of nono.

Song et al. [11] assessed the probiotic potential of *Pediococcus acidilactici* M76 for the lactic acid fermentation of black raspberry extract, whereas Thongwai et al. [12] identified a bacterial isolate from contaminated honey wine in a honey factory in northern Thailand, as belonging to the *Komagataeibacter maltaceti* species, and characterized its ability to produce bacterial cellulose.

Finally, Podrzaj et al. [13] compared the phenotypes of 12 *Clostridium tyrobutyricum* strains with different genotypic and proteotypic profiles, showing that strain-specific germination and growth characteristics should be considered among other factors to evaluate the risk of cheese spoilage by this spoilage agent of hard and semi-hard cheeses.
